# Screening of Antiviral Components of Yinhuapinggan Granule and Protective Effects of Yinhuapinggan Granule on MDCK Cells with Influenza A/H1N1 Virus

**DOI:** 10.1155/2022/1040129

**Published:** 2022-02-15

**Authors:** Tianhang Chen, Haixia Du, Huifen Zhou, Jiehong Yang, Jiaqi Zhu, Xin Tong, Yuting Yang, Jiayang Wan, Yichen Fan, Yiyu Lu, Yu He, Haitong Wan

**Affiliations:** ^1^School of Life Science, Zhejiang Chinese Medical University, Hangzhou 310053, China; ^2^College of Basic Medicine, Zhejiang Chinese Medical University, Hangzhou 310053, China; ^3^Department of Obstetrics and Gynecology, Peking University First Hospital, Beijing 100034, China; ^4^Institute of Microbiology, Zhejiang Center for Disease Control and Prevention, Hangzhou 310009, China; ^5^College of Pharmaceutical Science, Zhejiang Chinese Medical University, Hangzhou 310053, China

## Abstract

**Background:**

Traditional Chinese medicine Yinhuapinggan granule (YHPG) has been used for treating upper respiratory tract infection like influenza, cough, and viral pneumonia. However, its active ingredients that really exert the main efficacy have not been well elucidated. This study is aimed at screening its antiviral components and investigating the potential therapeutic mechanisms of YHPG against the influenza A/PR8/34 (H1N1) virus in Madin Darby canine kidney (MDCK).

**Methods:**

MDCK cells were infected with the influenza virus and then treated with ribavirin, YHPG, and main active ingredients in YHPG. Based on the maximum nontoxic concentration (TC_0_), half-maximal toxic concentration (TC_50_), half-maximal inhibitory concentration (IC_50_), and therapeutic index (TI), interferon-*β* (IFN-*β*) and interleukin-6 (IL-6) levels were measured using enzyme-linked immunosorbent assay (ELISA), and the gene expression of TLR7, MyD88, tumor necrosis factor receptor-associated factor 6 (TRAF6), c-Jun amino terminal kinase (JNK), p38 mitogen-activated protein kinase (p38 MAPK), and p65 nuclear transcription factor-kappa B (p65 NF-*κ*B) was quantified using reverse transcription-polymerase chain reaction (RT-PCR).

**Results:**

The results indicated that the components of YHPG, such as ephedrine hydrochloride, pseudoephedrine hydrochloride, chlorogenic acid, and emodin, had significant antiviral effects. High and medium doses of YHPG effectively reduced the cytopathic effect (CPE) and significantly decreased IFN-*β* and IL-6 levels in the supernatant. Simultaneously, the transcript levels of TLR7, MyD88, TRAF6, JNK, p38 MAPK, and p65 NF-*κ*B decreased in infected MDCK cells. Moreover, a certain dose-dependent relationship among different groups of YHPG was observed.

**Conclusions:**

These results indicated that YHPG and the components of YHPG had a significant inhibitory function on the proliferation of the H1N1 virus. The mechanism might be associated with suppressing the activation of the TLR7/MyD88 signaling pathway, a decrease in the mRNA expression of key target genes, and inhibition of IFN-*β* and IL-6 secretion.

## 1. Introduction

Influenza, which has a high incidence, extensive prevalence, and rapid dissemination, is an acute respiratory tract infection caused by the influenza virus of the family Orthomyxoviridae. The virus can be divided into the A, B, and C types based on the nucleoprotein's antigenic characteristics. The influenza A virus tends to undergo antigenic drift, causing worldwide pandemics and seriously threatening human health. Current drug treatment for viruses mainly includes M2 ion channel and neuraminidase inhibitors such as amantadine and oseltamivir [1, 2]. Despite their availability, these drugs often fail quickly due to surface antigen variation. Compared with synthetic chemical drugs, traditional Chinese medicines (TCM) against the influenza virus have the advantages of multiple targets and insignificant adverse side effects [3]. Therefore, the development and use of natural medicine and related mechanisms are necessary.

Yinhuapinggan granule (YHPG), formerly named Jin Pinggan, is based upon the classical Ephedra decoction formula and was improved by the clinical experience of Professor Wan Haitong. It is mainly composed of *Flos Lonicerae Japonicae*, *Herba Ephedrae*, *Puerariae Lobatae Radix*, *Polygoni Cuspidati Rhizoma*, *Armeniacae Semen Amarum*, and *Glycyrrhizae Radix*, with a ratio of 4 : 4 : 4 : 2 : 2 : 1 (Table 1). The high-performance liquid chromatography (HPLC) analysis of YHPG has been performed using an established method in our lab (Supplementary Figure 1) [4]. Clinical trials show that YHPG had good clinical efficacy and safety [5, 6]. Previous studies have shown that YHPG significantly inhibited influenza virus replication in chicken embryos *in vitro* [7]. *In vivo*, YHPG was reported to relieve lung tissue injury in mice infected with the influenza A virus [8] and to possess antitussive [9], anti-inflammatory, and analgesic [10] properties. YHPG also reduced and regulated immune functions in mice, which might be associated with the regulation of Toll-like receptor (TLR) signaling pathways [4]. Thus, to elucidate the mechanism of YHPG-induced anti-influenza viral activity, this study observed the *in vitro* antiviral effect of YHPG in MDCK cells by examining the levels of interferon-*β* (INF-*β*) and interleukin-6 (IL-6), as well as the expression levels of target genes of TLR7, MyD88, TRAF6, JNK, p38 MAPK, and p65 NF-*κ*B in MDCK cells infected with influenza virus. Simultaneously, we should understand the anti-influenza virus components in YHPG. This study may provide an experimental basis for further systematic research on YHPG that may promote its clinical use.

## 2. Materials and Methods

### 2.1. Drugs and Reagents

**Figure 1:**
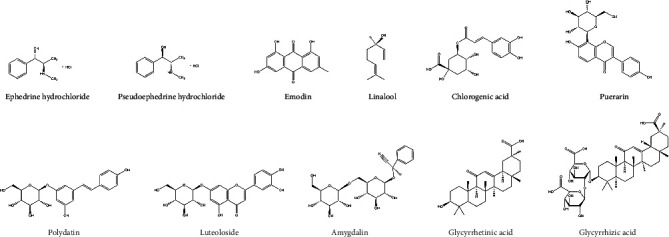
The chemical structures of eleven major components in YHPG.

*Flos Lonicerae Japonicae*, *Herba Ephedrae*, *Puerariae Lobatae Radix*, *Polygoni Cuspidati Rhizoma*, *Armeniacae Semen Amarum*, and *Glycyrrhizae Radix* were purchased from Hangzhou Huadong Chinese Herbal Medicine Co., Ltd. and were identified by Prof. Shengwu Huang, College of Pharmaceutical Science, Zhejiang Chinese Medical University, where voucher specimens were deposited (Table 1). The crude slices of these drugs were in conformity with the quality standards of Chinese Pharmacopoeia (2020 edition). Ribavirin granules were obtained from Sichuan Baili Pharmaceutical Co., Ltd. Glycyrrhizic acid, puerarin, chlorogenic acid, luteoloside, polydatin, amygdalin, emodin, glycyrrhetinic acid, and linalool were purchased from Nanjing Shizhou Biotechnology Co., Ltd. (Nanjing, China). Ephedrine hydrochloride and pseudoephedrine hydrochloride were obtained from the National Institute for Food and Drug Control of China (Beijing, China). All standard substances had more than 98% purity. Modern pharmacological studies have shown that eleven components are the main active components and are present in YHPG with a relatively high content (their chemical structures are shown in Figure 1) [4]. Minimum essential medium (MEM) culture medium, phosphate-buffered saline (PBS), penicillin and streptomycin stock solutions, fetal bovine serum (FBS), and 0.25%-EDTA trypsin were all purchased from Gibco (California, USA). The reverse transcription reagent kit and SYBR Premix Ex Taq™ II reagent kit were obtained from TaKaRa Bio Inc. (Kusatsu, Japan). The RNA extraction reagent kit was purchased from Qiagen (Hilden, Germany). The interferon-*β* (IFN-*β*) and interleukin-6 (IL-6) enzyme-linked immunosorbent assay (ELISA) reagent kits were obtained from Shanghai FanKe Biological Technology Co., Ltd. (Shanghai, China).

### 2.2. Virus Strain and Cell Lines

Influenza A/PR8/34 (H1N1) virus was provided by the Zhejiang Provincial Center for Disease Control and Prevention. After amplification in 9-day-old chicken embryos, the virus was stored at -80°C for future use. The viral titer was 1 : 1,024, as determined using the hemagglutination test. MDCK cells were cultivated in MEM containing 10% heat-inactivated FBS, 1% 2 mM Lg, and 1% PS in a humidified atmosphere containing 5% CO_2_ at 37°C.

### 2.3. Measurement of Viral Infectivity Titers

To obtain nine concentrations of viral solutions (10^−1^-10^−9^), influenza virus A/PR8/34 (H1N1) was serially diluted 10-fold in viral growth medium. A 100 *μ*L aliquot of these viral solutions was added to a well of a 96-well plate containing a monolayer of confluent MDCK cells, with six replicate wells for each concentration. Meanwhile, a control group with a cell maintenance solution was included. The cells were cultured in an incubator at 37°C and 5% CO_2_ and observed every day under an inverted microscope to evaluate the cytopathic effect (CPE). The 50% tissue culture infective dose (TCID_50_) was calculated using the Reed-Muench method.

### 2.4. Measurement of Drug Cytotoxicity

YHPG and its components (ephedrine hydrochloride, pseudoephedrine hydrochloride, glycyrrhizic acid, puerarin, chlorogenic acid, luteoloside, polydatin, amygdalin, emodin, glycyrrhetinic acid, and linalool) and ribavirin granules (positive drug) were serially diluted 2-fold in the cell maintenance solution. MDCK cells at the logarithmic growth phase were inoculated into 96-well plates and cultured at 37°C and 5% CO_2_. One hundred *μ*L aliquot of YHPG at 10 concentrations, from 10 to 0.020 mg·mL^−1^, ephedrine hydrochloride from 125 to 0.98 *μ*g·mL^−1^, pseudoephedrine hydrochloride from 125 to 0.98 *μ*g·mL^−1^, glycyrrhizic acid from 1,000 to 7.81 *μ*g·mL^−1^, puerarin from 1,000 to 7.81 *μ*g·mL^−1^, chlorogenic acid from 1,000 to 7.81 *μ*g·mL^−1^, luteoloside from 1,000 to 7.81 *μ*g·mL^−1^, polydatin from 2,000 to 15.63 *μ*g·mL^−1^, amygdalin from 250 to 1.96 *μ*g·mL^−1^, emodin from 125 to 0.98 *μ*g·mL^−1^, glycyrrhetinic acid from 1,000 to 7.81 *μ*g·mL^−1^, and linalool from 2,000 to 15.63 *μ*g·mL^−1^ at eight concentrations, and ribavirin granule at 10 concentrations, from 25 to 0.050 mg·mL^−1^, were added to each well. Each concentration was repeated six times, and a control group with cell maintenance solution was included. After the cells were routinely incubated for 48 h, 20 *μ*L of 5 mg·mL^−1^ 3-(4,5-dimethylthiazol-2-yl)-2,5-diphenyltetrazolium bromide (MTT) solution was added, and the cells were cultured for another 4 h. The supernatant was discarded, and 150 *μ*L/well dimethyl sulfoxide (DMSO) was added; the cells were then shaken at low speed for 15 min on a shaker until the crystallized deposits were completely dissolved. The absorbance value of each well at 490 nm was measured using a microplate reader. Cell survival and inhibition rates were calculated according to the following formulae:

(1) $$\begin{array}{c} \text{Cell survival rate }\left(\%\right)=\left(\text{average absorbance value of the drug group}/\text{average absorbance value of the control group}\right)\times 100\%, \end{array}$$

(2) $$\begin{array}{c} \text{Cell inhibition rate }\left(\%\right)=100\%-\text{cell survival rate}. \end{array}$$

In addition, the half-maximal toxic concentration (TC_50_) and the maximum nontoxic concentration (TC_0_) were calculated using the Reed-Muench method.

### 2.5. Antiviral Mode of Action

#### 2.5.1. Preventive Function

Starting from the maximum nontoxic concentration, the YHPG (0.625 mg·mL^−1^, 0.3125 mg·mL^−1^, and 0.156 mg·mL^−1^) and ribavirin (0.780 mg·mL^−1^, 0.390 mg·mL^−1^, and 0.195 mg·mL^−1^) solutions were serially diluted 2-fold in cell maintenance solution. After reaching confluent monolayers in 96-well plates, the supernatant was discarded, and the cells were washed twice with PBS; 100 *μ*L of the different drug concentrations was added to each well, with six replicate wells for each concentration. Control (with cell maintenance solution only) and mock groups (with the virus only) were included. After incubation at 37°C and 5% CO_2_ for 1 h, the drug solution was discarded, and 100 *μ*L of the diluted virus at 10 TCID_50_ was added. After adsorption at 37°C and 5% CO_2_ for 1 h, the supernatant was discarded, and the MEM cell maintenance solution was added.

#### 2.5.2. Therapeutic Effects

The diluted virus at 10 TCID_50_ was added to a 96-well plate. After adsorption for 1 h, 100 *μ*L of different concentrations of prediluted YHPG or ribavirin solution was added to each well, with six replicate wells for each concentration. Control and mock groups were set up.

For the two experiments described above, CPE was observed every day under an inverted microscope. When CPE in the mock group reached 75%, absorbance at 490 nm was assessed using the MTT method to calculate cell survival rates, the antiviral effect of the drugs, the half-maximal inhibitory concentration (IC_50_), and the therapeutic index (TI).

Antiviral effectiveness of drugs (%) = (average absorbance value of the drug group − average absorbance value of the mock group)/(average absorbance value of the control group − average absorbance value of the mock group) × 100%.

TI = TC_50_/IC_50_.

### 2.6. Screening of Active Components from YHPG against Influenza A (H1N1) Virus

Starting from TC_0_, YHPG and its components (ephedrine hydrochloride, pseudoephedrine hydrochloride, glycyrrhizic acid, puerarin, chlorogenic acid, luteoloside, polydatin, amygdalin, emodin, glycyrrhetinic acid, and linalool) were diluted to five concentrations of drug-containing solution under maximum nontoxic concentration, and ribavirin was diluted to 780 *μ*g·mL^−1^. The MDCK cells were cultured on 96-well plates until they grew into a monolayer; the cells were then inoculated with 10 TCID_50_ H1N1 virus, 100 *μ*L/well, adsorbed in a 5% CO_2_ incubator at 37°C for 1 h, and then washed with PBS three times. After adding the above concentration of drugs, the cells were kept in culture with 100 *μ*L/well and six replicate wells for each concentration. Control and mock groups were set up. After 48 h, the CCK-8 method was used to calculate the antiviral effectiveness of the drugs.

### 2.7. Analysis of Viral Load by RT-PCR

The drug concentration with high antiviral efficiency under the TC_0_ was selected for the experiment. The experiment was divided into 12 groups: a control group, the mock group, the control+YHPG group (625 *μ*g·mL^−1^), the ribavirin group (780 *μ*g·mL^−1^), the YHPG high-dose group (625 *μ*g·mL^−1^), the YHPG medium-dose group (312.5 *μ*g·mL^−1^), the YHPG low-dose group (56.25 *μ*g·mL^−1^), the ephedrine hydrochloride group (62.5 *μ*g·mL^−1^), the pseudoephedrine hydrochloride group (62.5 *μ*g·mL^−1^), the chlorogenic acid group (31.25 *μ*g·mL^−1^), the luteoloside group (250 *μ*g·mL^−1^), and the emodin group (62.5 *μ*g·mL^−1^). The MDCK cells were seeded on 6-well plates with 5 × 10^5^/mL, 2.5 mL/well until the cells grew to 70%~80%. Except for the control group and the control+YHPG medium-dose group, the other groups were inoculated with 10 TCID_50_ virus diluent and placed in an incubator at 37°C, 5% CO_2_ for 1 h. The plates were gently shaken every 15 min. After adsorption for 1 h, the culture medium was discarded, PBS was added to wash the cells twice, and the above concentrations of drugs were added. The control group and mock group were added with the same volume of serum-free DMEM medium and grown in a cell incubator (Thermo Scientific, Waltham, MA, USA) in an atmosphere of 5% CO_2_ at 37°C for 24 h, and CPE was observed at 24 h. Then, the samples were stored at -80°C for RT-PCR analysis. The total RNA from the cells was extracted with RNeasy Mini (QIAGEN, Germany) and finally dissolved in 40 *μ*L of RNase-free water. RT-PCR assay was performed on cDNA samples *via* the SYBR Premix Ex Taq™ II (Takara, Dalian, China). The primer sequences that were used to amplify the influenza virus M1 gene (IFV-M1) and glyceraldehyde 3-phosphate dehydrogenase (GAPDH) (Table 2) were designed and synthesized by Sangon Biotech, Co., Ltd. (Shanghai, China). The reaction conditions were 95°C for 2 min, followed by 95°C for 15 s for 40 cycles of denaturation and extended at 55°C for 35 s. RT-PCR analysis was performed using an automatic thermocycler (QuantStudio 12K Flex Real-Time PCR System, Applied Biosystems Co., USA). The relative expression levels of the target genes were quantified using the 2^-*ΔΔ*Ct^ method and normalized using GAPDH as the internal control according to the following equation:

(3) $$\begin{array}{c} {}^{∆∆}\text{Ct}={\left(\text{C}{\text{t}}_{\text{target gene}}-\text{C}{\text{t}}_{\text{internal control}}\right)}_{\text{experimental group}}-{\left(\text{C}{\text{t}}_{\text{target gene}}-\text{C}{\text{t}}_{\text{internal control}}\right)}_{\text{control group}}. \end{array}$$

### 2.8. Detection of IFN-*β* and IL-6 Secretion Levels by ELISA

After growing to a confluent monolayer in plates, 2 mL/well of the diluted virus at 10 TCID_50_ was added. The cell maintenance solution was added to the control group. After viral adsorption for 1 h, the supernatant was discarded; diluted YHPG and ribavirin solutions were added to the drug treatment groups, whereas the virus growth medium was added to the mock group. The cells were incubated at 37°C and 5% CO_2_ for 24 h. After the cell supernatant was collected, the IFN-*β* and IL-6 secretion levels were measured using the ELISA kits according to the manufacturer's manual.

### 2.9. Detection of the Expression of Related Genes by RT-PCR

Diluted virus at 10 TCID_50_ was added (1 mL/flask) to confluent MDCK cells in a T25 cell culture flask. After virus adsorption for 1 h, the supernatant was discarded, and the cells were washed twice with PBS; different doses of YHPG (a high dose of 0.625 mg·mL^−1^, a medium dose of 0.313 mg·mL^−1^, and a low dose of 0.156 mg·mL^−1^) were then added. In addition, the control, mock, and ribavirin (0.780 mg·mL^−1^) groups were included. The cells were continuously cultured at 37°C and 5% CO_2_ for 24 h. Total RNA was extracted from each sample. cDNA synthesis and RT-PCR were performed. The operation methods were the same as Analysis of Viral Load by RT-PCR. All primers were synthesized by Shanghai Sangon Biotechnology Co. Ltd. The primer sequences are shown in Table 2. The threshold cycle (Ct) value of each sample was calculated using the relative quantitation method and glyceraldehyde 3-phosphate dehydrogenase (GAPDH) as the internal control. The relative mRNA expression levels in all groups were calculated using the 2^-∆∆Ct^ formula.

### 2.10. Statistical Analysis

SPSS19.0 statistical software was used. Experimental data were expressed as the mean ± standard deviation ($\bar{x}\pm s$), and comparison of measured data among groups was performed using one-way analysis of variance. Pairwise comparison among groups was performed using the least significant difference (LSD) *t-*test when variances were homogeneous; when variances were not homogeneous, the *Tamhane* test was used. *P* < 0.05 indicated a significant difference, and *P* < 0.01 indicated a highly significant difference.

## 3. Results

### 3.1. Measurement of Viral Infectivity Titers

According to the experimental results, the TCID_50_ of influenza A/PR8/34 (H1N1) virus was 10^–3.5^/0.1 mL, as calculated using the Reed-Muench method.

### 3.2. Measurement of Drug Cytotoxicity

The morphological changes of the MDCK cells were observed by a microscope. The cells became round, shrunk, and adhesive and the intercellular space increased. Some cells were detached. The TC_50_ and TC_0_ for YHPG, YHPG's components, and ribavirin granules are shown in Table 3.

### 3.3. Antiviral Mode of Action

**Figure 2:**
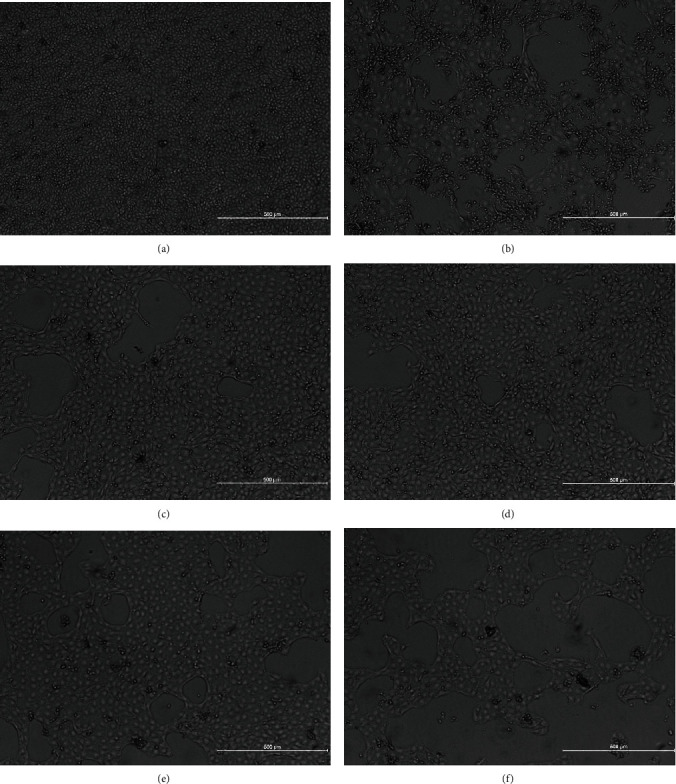
The preventive functions of YHPG and ribavirin granules against influenza virus: (a) control group; (b) mock groups; (c) ribavirin group (0.780 mg·mL^−1^); (d) high-dose YHPG group (0.625 mg·mL^−1^); (e) medium-dose YHPG group (0.313 mg·mL^−1^); (f) low-dose YHPG group (0.156 mg·mL^−1^).

The preventive function of YHPG and ribavirin granules against the influenza virus was not obvious, with antiviral effectiveness values of <30% (Table 4). Also, all MDCK cells exhibited CPE at all concentrations of YHPG (Figure 2).

**Figure 3:**
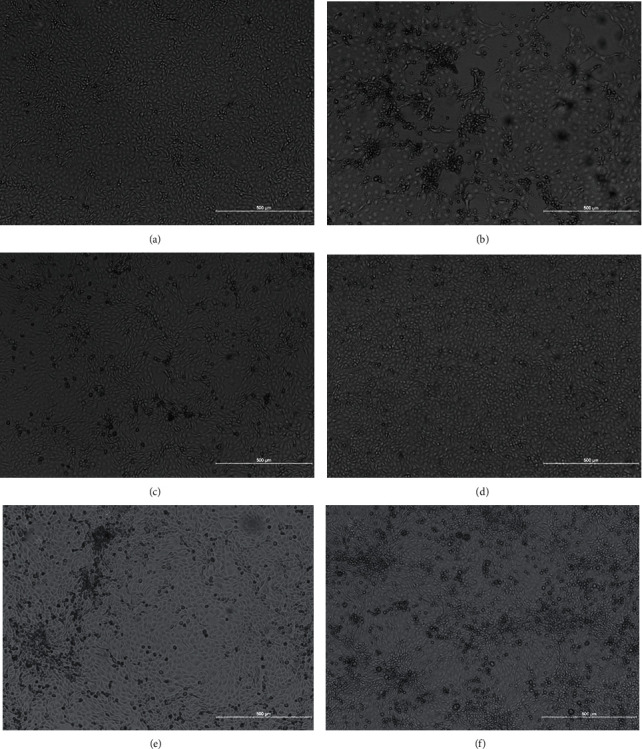
The therapeutic effect of YHPG and ribavirin granules on influenza virus: (a) control group; (b) mock groups; (c) ribavirin group (0.780 mg·mL^−1^); (d) high-dose YHPG group (0.625 mg·mL^−1^); (e) medium-dose YHPG group (0.313 mg·mL^−1^); (f) low-dose YHPG group (0.156 mg·mL^−1^).

YHPG at concentrations of 0.313 and 0.625 mg·mL^−1^ had obvious therapeutic effects on the influenza virus (Figure 3), with antiviral effectiveness > 90%, and CPE gradually increased with the drug concentration. At concentrations of 0.156-0.625 mg·mL^−1^ YHPG, the antiviral effectiveness was >50%, the same antiviral effectiveness achieved with 0.390-0.780 mg·mL^−1^ ribavirin. The antiviral effectiveness of ribavirin was >90% at 0.780 mg·mL^−1^. The specific IC_50_ and TI values are shown in Table 5.

### 3.4. Effect of YHPG and the Main Active Components on Influenza A/H1N1 Virus

YHPG and its active components (ephedrine hydrochloride, pseudoephedrine hydrochloride, glycyrrhizic acid, puerarin, chlorogenic acid, luteoloside, polydatin, amygdalin, emodin, glycyrrhetinic acid, and linalool) in different concentrations acted on MDCK cells infected with influenza A/H1N1 virus. The corresponding antiviral effective rates are shown in Table 6. We found that glycyrrhizic acid, puerarin, polydatin, amygdalin, glycyrrhetinic acid, and linalool had no obvious antiviral effect, and their antiviral efficiency was less than 60%. The antiviral effects of luteoloside and emodin groups were obvious, and the antiviral efficiency of the maximum nontoxic concentration was more than 60%. The antiviral effect of YHPG and ephedrine hydrochloride, pseudoephedrine hydrochloride, and chlorogenic acid groups was evident. Furthermore, there was a positive correlation between antiviral efficacy and drug concentration.

### 3.5. Effect of YHPG and the Main Active Components on CPE and Viral Load

**Figure 4:**
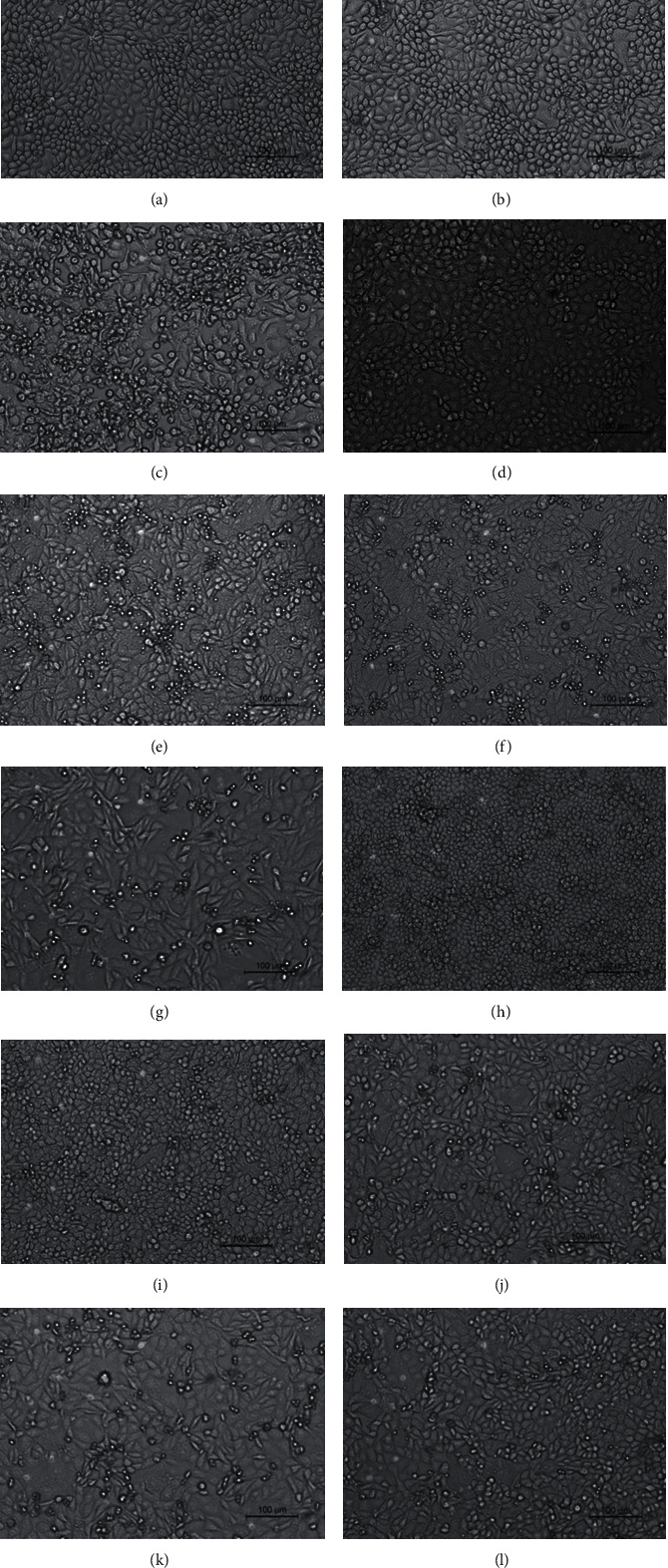
Effects of YHPG and the main active components on CPE of MDCK cells infected by influenza A/H1N1 virus (×10) after 24 h of treatment: (a) control group; (b) control+YHPG group (625 *μ*g·mL^−1^); (c) mock group; (d) ribavirin group (780 *μ*g·mL^−1^); (e) high-dose YHPG group (625 *μ*g·mL^−1^); (f) medium-dose YHPG group (312.5 *μ*g·mL^−1^); (g) low-dose YHPG group (156 *μ*g·mL^−1^); (h) ephedrine hydrochloride group (62.5 *μ*g·mL^−1^); (i) pseudoephedrine hydrochloride group (62.5 *μ*g·mL^−1^); (j) chlorogenic acid group (31.25 *μ*g·mL^−1^); (k) luteoloside group (250 *μ*g·mL^−1^); (l) emodin group (62.5 *μ*g·mL^−1^).

**Figure 5:**
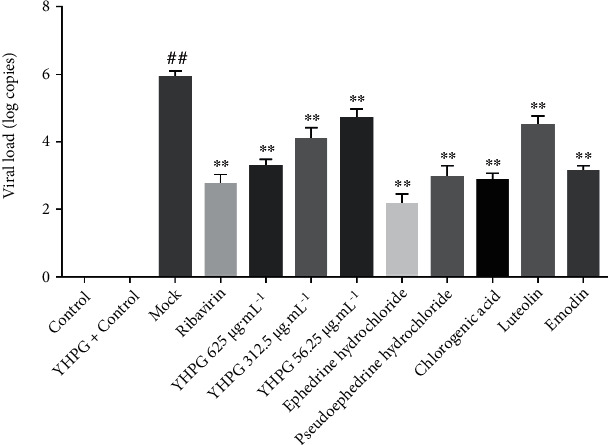
Viral load in H1N1 virus-infected MDCK cells following the treatment of YHPG and the main active components. Compared with the control group, ^##^*P* < 0.01; compared with the mock group, ^∗∗^*P* < 0.01.

When MDCK cells were infected with influenza A virus, the morphology of MDCK cells changed, and the cells shrunk and became detached, most of them adhering to each other in suspension at the surface of the medium; YHPG and its active components ephedrine hydrochloride, pseudoephedrine hydrochloride, chlorogenic acid, luteoloside, emodin, and ribavirin could significantly reduce the CPE (Figure 4). No viral RNA was detected in uninfected control cells (Figure 5). After H1N1 infection, the mock group's viral load was significantly higher than that of the control group. Compared with the mock group, the viral load in each dose group of YHPG, ephedrine hydrochloride, pseudoephedrine hydrochloride, chlorogenic acid, luteoloside, emodin, and ribavirin was significantly decreased after 24 h (*P* < 0.01). Moreover, after 24 h treatment, the decrease in the viral load in the YHPG 625 *μ*g·mL^−1^, ephedrine hydrochloride, pseudoephedrine hydrochloride, chlorogenic acid, emodin, and ribavirin was more obvious.

### 3.6. Detection of IFN-*β* and IL-6 Secretion Levels Using ELISA

**Figure 6:**
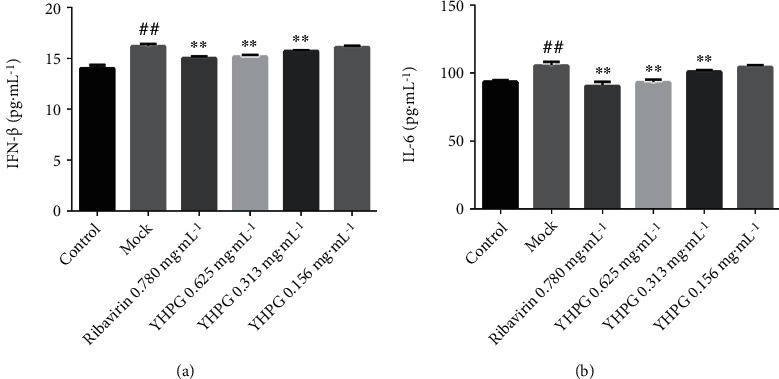
Effects of YHPG on the levels of IFN-*β* and IL-6 secreted by H1N1 virus-infected MDCK cells ($\bar{x}\pm s$, *n* = 9). Compared with the control group, ^##^*P* < 0.01; compared with the mock group, ^∗∗^*P* < 0.01.

The fitted regression equations of the control samples of IFN-*β* and IL-6 were *Y* = 64.119*X* − 3.8592 (*R*^2^ = 0.9969) and *Y* = 836.79*X* − 23.329 (*R*^2^ = 0.9974), respectively. Based on these equations, the levels of IFN-*β* and IL-6 in samples were measured (Figure 6). Compared with the control group, the secretion levels of IFN-*β* and IL-6 in the mock group significantly increased (*P* < 0.01). Compared with the mock group, the YHPG groups of high and medium doses all exhibited significantly decreased IFN-*β* and IL-6 levels (*P* < 0.01), whereas no significant difference was observed for the low-dose group. Also, there was a certain dose-dependent relationship among different YHPG groups.

### 3.7. Detection of the Expression of Related Genes in MDCK Cells Infected with the Influenza Virus Using RT-PCR

**Figure 7:**
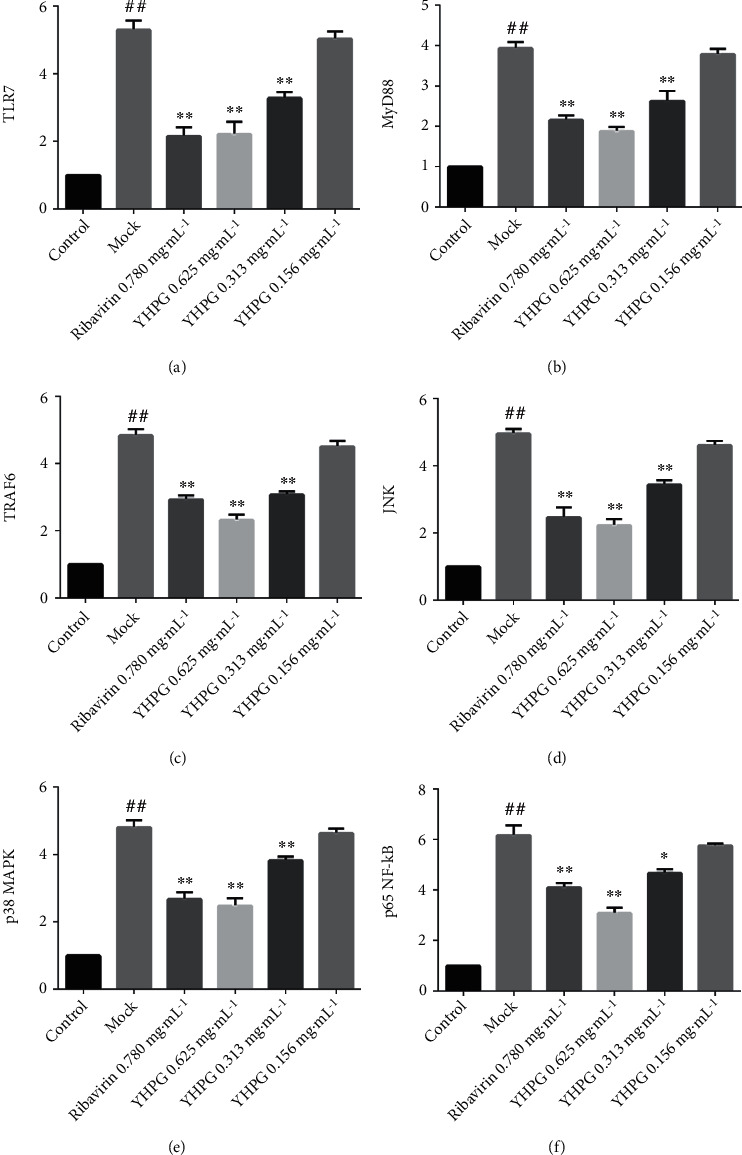
Effects of YHPG on the RNA expression of related genes in H1N1 virus-infected MDCK cells ($\bar{x}\pm s$, *n* = 9). Compared with the control group, ^##^*P* < 0.01; compared with the mock group, ^∗^*P* < 0.05 and ^∗∗^*P* < 0.01.

The levels of TLR7, MyD88, TRAF6, JNK, p38 MAPK, and p65 NF-*κ*B gene expression in the mock group were significantly upregulated compared with those in the control group (*P* < 0.01), suggesting that the influenza virus activated the TLR7 pathway (Figure 7). Compared with the mock group, the expression levels of these target genes in MDCK cells infected with influenza virus were significantly reduced in the high- and medium-dose YHPG groups (*P* < 0.01, *P* < 0.05), whereas no significant difference was found for the low-dose group (*P* > 0.05).

## 4. Discussion

The influenza virus is a respiratory pathogen that affects humans and causes severe morbidity and mortality. In this experiment, the effects of YHPG on MDCK cells infected by the H1N1 virus were investigated. YHPG can disperse wind and ventilate the lungs and clear away heat and toxic materials. Pharmacological studies have indicated that *Flos Lonicerae Japonicae* possesses antibacterial, antiviral, antipyretic, and anti-inflammatory properties and enhances immunity [11]. *Glycyrrhizae Radix* has anti-inflammatory, antiviral, and antitumor functions [12]. *Herba Ephedrae* has antioxidant and antiviral functions [13], and *Polygoni Cuspidati Rhizoma* has antibacterial, antiviral, and antitumor functions [14, 15]. From the literature, chlorogenic acid, an active component of *Flos Lonicerae Japonicae*, has shown an inhibitory effect on virus replication [16]. The main component of *Glycyrrhizae Radix*, glycyrrhizin, may protect mice exposed to a lethal amount of influenza virus through the stimulation of IFN-*γ* production by T cells and has been reported to inhibit influenza A virus uptake into the cell [17]. Glycyrrhizin concentrations that inhibited H5N1-induced proinflammatory gene expression did not affect natural killer cells' cytolytic activity [18]. Furthermore, emodin, an active component of *Polygoni Cuspidati Rhizoma*, inhibits the replication of influenza virus H1N1 in A549 cells [19]. So, we hypothesize that chlorogenic acid, glycyrrhizin, and emodin may be the active components of YHPG against the influenza virus. However, YHPG is composed of many traditional Chinese medicines and has more complex components. Additional investigations must identify the possible anti-influenza bioactive components in YHPG. Based on preliminary evidence, this study further confirmed that YHPG, within the effective concentration range, has a marked therapeutic effect on the influenza virus, effectively reducing CPE and has protective effects on MDCK cells after influenza virus infection. Simultaneously, we found that the main antiviral components of YHPG were ephedrine hydrochloride, pseudoephedrine hydrochloride, chlorogenic acid, and emodin.

TLRs comprise a group of important pathogen-related pattern recognition receptors (PPRs) [20]. These receptors specifically recognize different pathogen-associated molecular patterns (PAMPs) [21], activate intracellular signaling pathways, mediate corresponding immune responses and induce proinflammatory cytokines, chemokines, and interferons, and have important functions against pathogens.

There are two major antiviral immune pathways mediated by TLRs: MyD88-dependent and MyD88-independent pathways. TLR7 signaling belongs to the MyD88-dependent pathway and is located in the inner body membrane, which can identify the virus single-stranded RNA (ssRNA) [22]. Upon pathogen infection, TLR7 interacts with its corresponding ligands, and the intracellular Toll/interleukin-1 receptor homology (TIR) domain of activated TLR7 binds to and interacts with the carboxyl-terminus of MyD88 [23]. Activated MyD88 then induces the phosphorylation of members of the interleukin-1 receptor-associated kinase (IRAK) family to activate TRAF6 and transmit the signal to MAPK, JNK, and p65 NF-*κ*B [24], ultimately resulting in the secretion of many types of immune-associated cytokines and chemokines for the antiviral response, such as IFN-*β* and IL-6 [25, 26]. Previous studies have found that the expression of TLR7 was significantly upregulated in immune cells infected with the influenza A virus [27]. Furthermore, TLR7 signaling also induced downstream proinflammatory cytokines [28]. MyD88 is essential for proinflammatory cytokine production [29] and optimal protection against various pathogens, including viruses [30, 31]. TRAF6 has been identified as a signal transducer, resulting in the production of cytokines [32]. JNK is activated during many viral infections [33] and is required for polarized differentiation of T helper cells into Th1 cells [34]. T cell receptor-activated p38*α* and p38*β* MAPK are important and redundant positive regulators of T cell proliferation and inflammatory autoimmunity [35]. The activation of NF-*κ*B, as a marker of the proliferation of influenza virus in host cells [36], is also a key regulator of gene expression of inflammatory factors and plays an important role in the pathogenesis of anti-influenza virus infection [37].

Previous experiments have suggested that the pathogenesis and severity of influenza virus infection are associated with various levels of IFN-*β* and IL-6 [38]. Studies have shown that increased proinflammatory cytokines and mononuclear factors (including IL-6) were observed in the serum of infected patients and in infected mice's lungs, which associated with the pathogenesis and severity of influenza virus infection [39–41]. IFN-*β*, as an important cytokine, is involved in the body's immune regulation and could enhance natural killer cells (NK cells), macrophages, and T lymphocyte activity, thus improving the antivirus ability [42]. It also plays an important role in antivirus and immune regulation by inducing antiviral proteins' expression.

Our study showed a significant upregulation of the content of IFN-*β* and IL-6 in cell supernatants and the levels of TLR7, MyD88, TRAF6, JNK, p38 MAPK, and p65 NF-*κ*B gene expression after MDCK cells were infected with influenza virus (*P* < 0.01), suggesting that the influenza virus infection activated the TLR7-MyD88 pathway, induced an inflammatory reaction, and caused the production of cytokines and chemokines in MDCK cells. The content of IFN-*β* and IL-6 in the cell supernatant and expression of cellular TLR7, MyD88, TRAF6, JNK, p38 MAPK, and p65 NF-*κ*B genes significantly decreased after influenza virus-infected MDCK cells were treated with high and medium doses of YHPG (*P* < 0.01, *P* < 0.05). CPE was also effectively reduced. Furthermore, compared with the mock group, MDCK treated with YHPG showed a dose-dependent manner. These results indicate that YHPG has a therapeutic effect on MDCK cells infected with the influenza virus. The mechanism of action may be associated with inhibition of the activation of TLR7/MyD88 signaling, thus inhibiting the secretion of IFN-*β* and IL-6.

Nonetheless, there are many subtypes of TLRs, and previous studies have indicated that YHPG could also exert antiviral function by inhibiting the activation of the TLR4/MyD88 pathway. The determination of whether YHPG exerts synergistic antiviral effects by regulating other subtypes requires further in-depth studies. Furthermore, it was reported that the tripartite motif-containing 29 (TRIM29) plays a critical role in host defense against influenza virus infection, a key adaptor in both the IRF-mediated type-I-interferon production and the NF-*κ*B-mediated proinflammatory signaling pathways [43]. Our previous study found that YHPG significantly downregulated the mRNA expression of IRF7 and the protein expressions of the phosphorylated forms of IRF3 in RAW264.7 cells [44, 45]. Therefore, TRIM29 will be another powerful biomarker for our in-depth and meticulous future studies.

In conclusion, this research examined the anti-influenza effect of YHPG *in vitro*, and the results indicated that YHPG and its main active components (ephedrine hydrochloride, pseudoephedrine hydrochloride, chlorogenic acid, and emodin) exerted antiviral function by inhibiting virus-related gene expression. A therapeutic effect of YHPG on MDCK cells infected with the influenza virus was observed. These results provide a theoretical basis for further clinical application of YHPG. In subsequent studies, we will further investigate the synergistic effect and compatibility mechanism of increasing effectiveness of the main active components in YHPG.

## Figures and Tables

**Table 1:** Component herbs of YHPG.

Pharmaceutical name	Botanical plant name	Family	Weight (g)	Used part
*Flos Lonicerae Japonicae*	*Lonicera japonica* Thunb.	Caprifoliaceae	10	Flower bud
*Herba Ephedrae*	*Ephedra sinica* Stapf.	Ephedraceae	5	Aerial part
*Puerariae Lobatae Radix*	*Pueraria lobata* (Willd.) Ohwi	Lamiaceae	10	Radix
*Polygoni Cuspidati Rhizoma*	*Polygonum cuspidatum* Sieb. et Zucc.	Polygonaceae	10	Root and rhizome
*Armeniacae Semen Amarum*	*Prunus armeniaca* L. var. ansu. Maxim.	Rosaceae	5	Fruit
*Glycyrrhizae Radix*	*Glycyrrhiza uralensis* Fischer	Leguminosae	2.5	Root and stolon

**Table 2:** Sequences of gene primers and fragments of products.

Target gene	Primer sequence (5′→3′)	Product length (bp)
M1	TCATTGGGATCTTGCACTTG	117
	ACTTTGGCACTCCTTCCGTA	
TLR7	TGCTCTGCTCTCTTCAACCAG	199
	CAATCACATGGGCCTTCGGA	
MyD88	CCAAGTAGGGTGGGCAGAAC	156
	CAAATGCTGGCATGTTGGGT	
TRAF6	CGTGTCACCCAGAGGTTCAG	200
	GTAGATCCCGTTGCACTGCT	
JNK	CTCCCCTTTGTCTTGCACTC	118
	TAACCCACAGCAGGGAAATC	
p38 MAPK	CTCGCACATGCCTACTTTGC	192
	ACAGAAACCAGGTGCTCAGG	
p65 NF-*κ*B	CAGCCATGGACGACCTGTTTC	113
	CTGCTTGGGTTGCTCGATGA	
GAPDH	GTGACACCCACTCTTCCACC	162
	GTGGTCCAGGAGGCTCTTAC	

**Table 3:** TC_50_ and TC_0_ of YHPG, active components, and ribavirin granules (*n* = 6).

Group	TC_50_ (mg·mL^−1^)	TC_0_ (mg·mL^−1^)
YHPG	0.970	0.625
Ribavirin granule	1.316	0.780
Ephedrine hydrochloride	8.260	0.0625
Pseudoephedrine hydrochloride	6.012	0.0625
Glycyrrhizic acid	6.375	0.0625
Chlorogenic acid	4.030	0.03125
Puerarin	3.076	0.125
Luteoloside	4.766	0.250
Polydatin	6.155	0.125
Amygdalin	7.636	0.0625
Emodin	2.901	0.0625
Glycyrrhetinic acid	6.863	0.0625
Linalool	15.433	0.0625

**Table 4:** The antiviral efficacy of different concentrations of YHPG and ribavirin on influenza A/H1N1 virus in preventive functions (*n* = 6).

Group	Drug concentration (mg·mL^−1^)	Antiviral efficiency (%)
Ribavirin granule	0.780	29.15 ± 2.04
YHPG	0.625	27.96 ± 2.55
	0.313	15.54 ± 2.56
	0.156	7.36 ± 2.82

**Table 5:** Inhibitory functions of YHPG and ribavirin granules on influenza A/H1N1 virus (*n* = 6).

Group	YHPG		Ribavirin granule	
	Therapeutic group	Preventive group	Therapeutic group	Preventive group
IC_50_ (mg·mL^−1^)	0.121	1.030	0.209	1.473
TI	8.020	0.940	6.300	0.890

**Table 6:** Comparison of antiviral efficacy of different concentrations of YHPG and its active components on influenza A/H1N1 virus.

Group	Drug concentration (*μ*g·mL^−1^)	Antiviral efficiency (%)	Group	Drug concentration (*μ*g·mL^−1^)	Antiviral efficiency (%)
YHPG	39.06	46.66 ± 3.69	Ephedrine hydrochloride	3.91	55.32 ± 2.59
	78.13	58.17 ± 2.72		7.81	65.34 ± 2.47
	156.25	67.43 ± 3.87		15.63	69.30 ± 2.63
	312.50	74.82 ± 1.85		31.25	86.90 ± 2.94
	625.00	86.41 ± 2.91		62.50	90.82 ± 3.92
Pseudoephedrine hydrochloride	3.91	62.84 ± 1.98	Chlorogenic acid	1.95	65.89 ± 2.14
	7.81	66.04 ± 3.15		3.91	70.29 ± 2.12
	15.63	81.13 ± 3.78		7.81	77.51 ± 3.46
	31.25	87.06 ± 2.47		15.63	80.22 ± 3.92
	62.50	90.80 ± 3.84		31.25	84.80 ± 1.88
Emodin	3.91	14.48 ± 1.84	Luteoloside	15.63	14.52 ± 3.99
	7.81	35.55 ± 2.47		31.25	27.46 ± 2.76
	15.63	50.80 ± 3.48		62.50	29.97 ± 3.46
	31.25	75.57 ± 2.29		125.00	47.29 ± 2.33
	62.50	87.09 ± 2.08		250.00	69.84 ± 3.26
Puerarin	7.81	35.48 ± 2.78	Polydatin	7.81	8.68 ± 2.87
	15.63	41.75 ± 3.22		15.63	12.96 ± 2.89
	31.25	48.93 ± 2.99		31.25	16.48 ± 1.95
	62.50	51.37 ± 2.88		62.50	28.90 ± 3.22
	125.00	59.72 ± 3.38		125.00	47.27 ± 2.38
Glycyrrhizic acid	3.91	6.01 ± 2.76	Amygdalin	3.91	9.59 ± 1.99
	7.81	7.54 ± 1.83		7.81	11.43 ± 2.74
	15.63	13.78 ± 2.81		15.63	19.46 ± 2.65
	31.25	24.25 ± 3.80		31.25	26.34 ± 3.47
	62.50	38.03 ± 3.77		62.50	29.29 ± 3.15
Glycyrrhetinic acid	3.91	8.81 ± 3.63	Linalool	3.91	3.42 ± 2.86
	7.81	10.85 ± 2.54		7.81	5.67 ± 3.05
	15.63	13.49 ± 2.97		15.63	9.46 ± 2.59
	31.25	16.21 ± 1.31		31.25	15.38 ± 3.39
	62.50	27.86 ± 1.10		62.50	23.85 ± 2.97
Ribavirin granule	780.00	92.57 ± 3.86			

## Data Availability

The data used to support the findings of this study are available from the corresponding author upon request.

## Supplementary Materials

Supplementary Materials Figure 1: 2D HPLC chromatograms of eight effective components in YHPG at 210 nm. 1: L-ephedrine; 2: D-pseudoephedrine; 3: chlorogenic acid; 4: amygdalin; 5: puerarin; 6: polydatin; 7: glycyrrhizic acid; 8: emodin.
