# Supplementary material for: Screening of Antiviral Components of Yinhuapinggan Granule and Protective Effects of Yinhuapinggan Granule on MDCK Cells with Influenza A/H1N1 Virus
Source: Biomed Res Int. 2022 Feb 15;2022:1040129. doi: 10.1155/2022/1040129 (PMC8863447; doi:10.1155/2022/1040129)
Supplement: Supplementary Materials — Figure 1: 2D HPLC chromatograms of eight effective components in YHPG at 210 nm. 1: L-ephedrine; 2: D-pseudoephedrine; 3: chlorogenic acid; 4: amygdalin; 5: puerarin; 6: polydatin; 7: glycyrrhizic acid; 8: emodin. [file 1040129.f1.docx]

**High-performance liquid chromatography (HPLC) analysis of Yinhuapinggan granule （YHPG）**

HPLC analysis was performed using an Agilent 1200 HPLC system, including a G1322A on-line degasser, a G1311A quaternary pump, a G1316A column oven, a G1329A autosampler, and a G1315B photodiode array detector, controlled by an Agilent Rev. B.04.01 chemstation. Chromatographic separation was performed on an Agilent Eclipse XDB-C18 column (4.6 × 250 mm, 5 μm) at 35 ℃. The photodiode array detector was set at 210 nm. The mobile phase was a mixture of water containing 0.1 % phosphoric acid (A) and acetonitrile (B). The linear gradients between the time points were 0–10 min at 3–9 % B, 10–25 min at 9–9 % B, 25–40 min at 9–20 % B, 40–65 min at 20–55 % B, 65–75 min at 55–70 % B, 75–80 min at 70–3 % B. The flow rate was maintained at 1.0 mL/min and the injection volume was 10 μL.


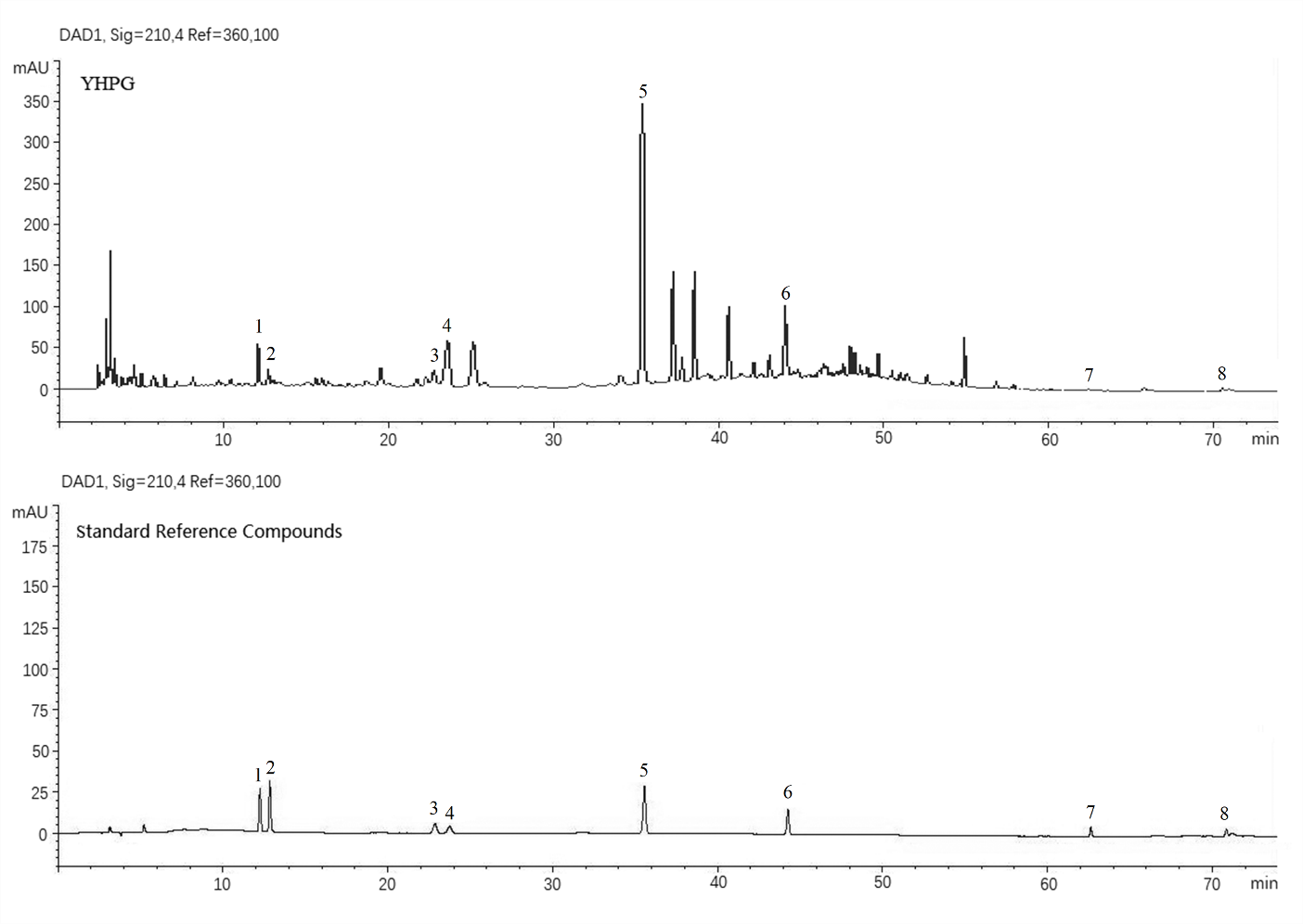


Fig. 1 2D HPLC chromatograms of eight effective components in YHPG at 210 nm. 1. L-ephedrine; 2. D-Pseudoephedrine; 3. Chlorogenic acid; 4. Amygdalin; 5. Puerarin; 6. Polygonin; 7. Glycyrrhizic acid; and 8. Emodin
